# Mechanisms of preferential bone formation in myeloma bone lesions by proteasome inhibitors

**DOI:** 10.1007/s12185-023-03601-2

**Published:** 2023-04-11

**Authors:** Emiko Nakaue, Jumpei Teramachi, Hirofumi Tenshin, Masahiro Hiasa, Takeshi Harada, Asuka Oda, Yusuke Inoue, So Shimizu, Yoshiki Higa, Kimiko Sogabe, Masahiro Oura, Tomoyo Hara, Ryohei Sumitani, Tomoko Maruhashi, Hiroki Yamagami, Itsuro Endo, Eiji Tanaka, Masahiro Abe

**Affiliations:** 1 Department of Orthodontics and Dentofacial Orthopedics, Tokushima University Graduate School of Biomedical Sciences, Tokushima, Japan; 2 Department of Oral Function and Anatomy, Graduate School of Medicine Dentistry and Pharmaceutical Sciences, Okayama University Graduate School, 2-5-1 Shikata, Okayama, 700-8525 Japan; 3 Department of Hematology, Endocrinology and Metabolism, Tokushima University Graduate School of Biomedical Sciences, 3-18-15 Kuramoto, Tokushima, 770-8503 Japan; 4 Department of Bioregulatory Sciences, Tokushima University Graduate School of Biomedical Sciences, Tokushima, Japan

**Keywords:** Osteoblast, Osteoclast, Proteasome inhibitor, Pulsatile treatment, Multiple myeloma

## Abstract

Proteasome inhibitors (PIs) can preferentially restore bone in bone-defective lesions of patients with multiple myeloma (MM) who respond favorably to these drugs. Most prior in vitro studies on PIs used continuous exposure to low PI concentrations, although pharmacokinetic analysis in patients has shown that serum concentrations of PIs change in a pulsatile manner. In the present study, we explored the effects of pulsatile treatment with PIs on bone metabolism to simulate in vivo PI pharmacokinetics. Pulsatile treatment with bortezomib, carfilzomib, or ixazomib induced MM cell death but only marginally affected the viability of osteoclasts (OCs) with F-actin ring formation. Pulsatile PI treatment suppressed osteoclastogenesis in OC precursors and bone resorption by mature OCs. OCs robustly enhanced osteoblastogenesis in cocultures with OCs and MC3T3-E1 pre-osteoblastic cells, indicating OC-mediated coupling to osteoblastogenesis. Importantly, pulsatile PI treatment did not impair robust OC-mediated osteoblastogenesis. These results suggest that PIs might sufficiently reduce MM cell-derived osteoblastogenesis inhibitors to permit OC-driven bone formation coupling while suppressing OC differentiation and activity in good responders to PIs. OC-mediated coupling to osteoblastogenesis appears to be a predominant mechanism for preferential occurrence of bone regeneration at sites of osteoclastic bone destruction in good responders.

## Introduction

Various novel anti-multiple myeloma (MM) agents have been developed. Nevertheless, repeated relapses and subsequent bone loss persist in patients with MM. MM cells stimulate bone resorption by enhancing osteoclastogenesis while suppressing bone formation by inhibiting osteoblastic differentiation from bone marrow stromal cells. Thus, MM causes extensive bone destruction and rapid bone loss [1–3]. In normal bone remodeling, homeostasis is tightly regulated by intercellular communication between osteoclasts (OCs) and osteoblasts (OBs) within the basic multicellular units of bone remodeling compartments [4]. OCs resorb damaged and old bone. In normal bone remodeling, OCs resorb bone and secrete coupling factors that recruit osteoblast precursors to the bone remodeling compartments, enhance osteoblastogenesis, and replace resorbed bone with new bone matrix [5]. In this manner, skeletal structure and integrity are maintained throughout life. In MM, however, this process is dysregulated. Besides MM cells enhance osteoclastic bone resorption while suppressing osteoblastic differentiation and, by extension, bone formation through factors such as soluble Wnt inhibitors that are secreted by the MM cells and are derived from their surrounding microenvironment [6–9]. In MM bone lesions, then, bone remodeling is skewed towards an increase in OC number and activity and the disruption of OC-mediated bone formation in the bone remodeling compartments.

Proteasome inhibitors (PIs) are major backbone drugs in MM treatment [10]. Proteasomes control the equilibrium between protein synthesis and degradation and maintain cellular function and survival. Proteasome inhibition results in the accumulation of misfolded and functional proteins in the lumen of the endoplasmic reticulum (ER) and the cytosol, thereby resulting in ER overload, reactive oxygen species (ROS) overproduction, functional intracellular protein disorders, and apoptosis in MM cells [11–13].

During PI treatment, bone formation is restored in the bone-destructive lesions of patients that respond favorably to these drugs [14–17]. Therefore, tumor reduction might trigger the anabolic effects of PIs by reducing MM cell-derived bone formation inhibitors. Unlike other novel anti-MM agents, PIs apparently induce robust bone formation preferentially in the bone-defective lesions that appear in radiographic images. However, the underlying mechanisms by which PIs mediate preferential bone recovery in the bone lesions of MM remain unknown.

Most prior in vitro studies on PIs consisted of long-term continuous exposures to low concentrations of PIs. Nevertheless, this approach does not accurately reflect the serum pharmacokinetic profile of PIs in human patients actually being administered these drugs [18–20]. In the present study, we endeavored to simulate the in vivo pharmacokinetics of PIs in human patients by examining the impact of pulsatile treatment at high PI concentrations on bone metabolism. We showed that pulsatile PI treatment suppressed osteoclastogenesis in OC precursors and bone resorption by mature OCs. By contrast, pulsatile PI treatment did not reduce OC viability although it promoted MM cell death. To mimic the bone remodeling, we first prepared mature OCs in vitro, and then MC3T3-E1 pre-osteoblastic cells were added to coculture with mature OCs; osteoblastogenesis of MC3T3-E1 cells was robustly enhanced in the presence of OCs, indicating OC-mediated coupling to osteoblastogenesis. The robust osteoblastogenesis of MC3T3-E1 cells was similarly observed upon pulsatile PI treatment after cocultures with OCs as well as in cocultures with pulsatile PI-pretreated OCs. These results suggest that pulsatile PI treatment suppress OC differentiation and activity while retaining OC’s potential to stimulate bone formation, namely OC-mediated coupling to osteoblastogenesis. OC-driven osteoblastogenesis might be a major mechanism by which bone is rebuilt in the bone-defective lesions where OCs reside in MM patients who respond rapidly and favorably to PIs. Therefore, OC-driven osteoblastogenesis, suppression of bone resorption by OCs, and the removal of osteoblastogenesis inhibitors via MM tumor reduction might cause preferential restoration of bone formation rather in osteoclastic bone destructive lesions in patients treated with PIs.

## Materials and methods

### Reagents

The following reagents were purchased from the indicated manufacturers: rabbit antibodies against c-Fos, IκBα, RelA, integrin β3, Sphingosine-1-phosphate (S1P); horseradish peroxidase-conjugated anti-rabbit and anti-mouse IgG (Cell Signaling Technology, Beverly, MA); mouse antibody against NFATc1, ephrinB2, cadherin 11 (CDH11, OB-cadherin) (Santa Cruz Biotechnology, Dallas, TX); rabbit antibody against p84, cathepsinK, Osterix/Sp7 (Abcam, Cambridge, UK); mouse antibody against β-actin (Sigma–Aldrich, St. Louis, MO); recombinant human M-CSF, β-glycerophosphate, ascorbic acid, and bortezomib (BTZ) (Cell Signaling Technology); carfilzomib (CFZ) (Chemietek, Indianapolis, IN); MLN2238 (ixazomib) (Karebay Biochem, Monmouth Junction, NJ); recombinant human BMP-2 (R&D Systems, Minneapolis, MN); and human-soluble receptor activator of NF-κB ligand (RANKL) (Oriental Yeast, Shiga, Japan).

### MM cells and cell culture

The human MM cell line MM.1S was obtained from the American Type Culture Collection (ATCC; Rockville, MD). The human MM cell line INA-6 was kindly provided by Renate Burger of the University of Kiel, Kiel, Germany. The mouse MM cell line 5TGM1 was kindly provided by Gregory R. Mundy of the Vanderbilt Center for Bone Biology, Vanderbilt University, Nashville, TN. All cells were cultured in RPMI 1640 medium (Sigma–Aldrich) supplemented with 10% (v/v) FBS and 50 mg/mL of penicillin/streptomycin (Thermo Fisher Scientific, Waltham, MA). The murine pre-osteoclastic cell line (RAW264.7) and the pre-osteoblastic cell line (MC3T3-E1) were purchased from ATCC and cultured in α-MEM (Sigma-Aldrich) supplemented with 10% (v/v) FBS and 50 mg/mL of penicillin/streptomycin.

### Osteoclast (OC) differentiation

OCs were produced from the murine pre-osteoclastic cell line RAW264.7 [21] or from mouse bone marrow cells as previously described [22]. The RAW264.7 cells (2 × 10^4^/mL) were cultured in M-CSF (10 ng/mL) and RANKL (50 ng/mL) for 4 days to generate mature OCs. Whole bone marrow cells were harvested from the femurs of C57BL/6 J mice (SLC, Tokyo, Japan). Nonadherent bone marrow cells (1 × 10^5^/mL) were collected and cultured in M-CSF (10 ng/mL) for 3 days to generate primary bone marrow-derived macrophages (BMMs) which were then cultured for 7–10 days in M-CSF (10 ng/mL) and RANKL (50 ng/mL) to generate mature OCs. The culture medium was changed every 2 days. The cells were fixed with 10% (v/v) neutral-buffered formalin. Tartrate-resistant acid phosphatase (TRAP)-positive cells were detected with a leukocyte acid phosphatase assay kit (Wako Pure Chemical, Osaka, Japan). TRAP-positive cells were observed under a light microscope (BZ-X800; Keyence, Osaka, Japan). Those containing ≥ 3 nuclei were scored as OCs. To investigate the effects of proteasome inhibitors (PIs) on osteoclastogenesis, primary BMMs and RAW264.7 cells were either untreated or subjected to BTZ (200 nM) or CFZ (500 nM) for 1 h or MLN2238 (200 nM) for 4 h. After the pulsatile PI treatment, the cells were washed twice with phosphate-buffered saline (PBS) and then cultured with M-CSF (10 ng/mL) and RANKL (50 ng/mL) for 4–7 days. OC differentiation was assessed by enumerating the multinucleate TRAP-positive cells. All mouse experiments were performed under the regulation and with the permission of the Animal Care and Use Committee of the Tokushima University, Tokushima, Japan (certificate No. T2022-13).

### Cell viability

Cell viability was determined by cell counting kit-8 (CCK-8) assay (Dojindo, Kumamoto, Japan) according to the manufacturer’s instructions. The MM cells, BMMs, mature primary OCs, RAW264.7 cells and MC3T3-E1 cells were cultured in 96-well plates containing BTZ (200 nM) or CFZ (500 nM) for 1 h, MLN2238 (200 nM) for 4 h. The absorbance of each well was measured at 450–655 nm in an iMark microplate reader (Bio-Rad Laboratories, Hercules, CA).

### Bone resorption assay

The effect of the PIs on RANKL-induced bone resorption was analyzed with a bone resorption assay kit in 48-well plates coated with fluorescein-labeled calcium phosphate (PG Research, Tokyo, Japan) as previously described [23]. Equal numbers of OCs generated from primary BMMs were seeded onto the assay plates and cultured with M-CSF (10 ng/mL) and RANKL (50 ng/mL) overnight. The OCs were treated with BTZ (200 nM) or CFZ (500 nM) for 1 h, MLN2238 (200 nM) for 4 h. The cells were then washed with PBS and further cultured in phenol red free α-MEM with M-CSF (10 ng/mL) and RANKL (100 ng/mL) for 48 h. The culture supernatants were collected, and calcium phosphate fluorescence intensity was measured in a microplate reader (SpectraMax i3; Molecular Devices, LLC, San Jose, CA). Bone resorption activity was determined by measuring the resorbed area with ImageJ software (National Institutes of Health, Bethesda, MD; http://imagej.nih.gov/ij/).

### F-actin ring staining

RAW264.7 cells were cultured in RANKL (50 ng/mL) to generate mature OCs which were then treated with BTZ (200 nM) or CFZ (500 nM) for 1 h, MLN2238 (200 nM) for 4 h. They were washed with PBS and cultured in RANKL (50 ng/mL) for 24 h. The OCs were fixed and stained with Acti-stain™ 488 phalloidin (Cytoskeleton, Denver, CO) following the manufacturer’s instructions and observed under a fluorescence microscope (BZ-X800; Keyence). DAPI (Thermo Fisher Scientific) was used to stain the nuclei of cells.

### OB differentiation

MC3T3-E1 cells were cultured in osteoblastic media (10 ng/mL BMP-2, 10 mM β-glycerophosphate, and 50 µg/mL ascorbic acid in 10% (v/v) FBS containing α-MEM). To examine OB differentiation, the cells were fixed with 10% (v/v) neutral-buffered formalin and visualized with an alkaline phosphatase staining kit (Wako Pure Chemical). The scanned images were analyzed with ImageJ software to measure the alkaline phosphatase (ALP)-positive areas.

### Co-culture experiments

Two types of experiments with pulsatile PI treatment were performed as follows: 1) Primary mature OCs prepared culture wells were first treated with or without BTZ (200 nM) or CFZ (500 nM) for 1 h or MLN2238 (200 nM) for 4 h. They were then washed twice with PBS, and MC3T3-E1 cells (1 × 10^5^/well) were seeded and cocultured with the OCs in osteoblastic media for 2 days. 2) MC3T3-E1 cells were first seeded onto primary mature OCs and both were treated with or without BTZ (200 nM) or CFZ (500 nM) for 1 h or MLN2238 (200 nM) for 4 h. The cells were then washed with PBS, and then cultured in osteoblastic media for 2 days. OB differentiation was assessed by ALP staining.

### Western blot analysis

Cells were collected and digested in RIPA lysis buffer (Santa Cruz Biotechnology). For cytosolic and nuclear preparation, the cells were lysed in NE-PER extraction reagent (Thermo Fisher Scientific) according to the manufacturer’s instructions. Western blot analysis was done with equal protein amounts of cell lysate, as described previously [24].

### Statistical analysis

Pairwise data comparisons were made with Student’s *t*-test. For multiple comparisons, statistical differences were determined by one-way analysis of variance (ANOVA) followed by Tukey’s test. *P* < 0.05 indicated a significant difference. All statistical analyses were performed with Statcel v. 4 software (OMS Publishing, Saitama, Japan).

## Results

### Proteasome inhibitor (PI) pulse treatment induced cell death in MMs but not OCs

Pharmacokinetic profiles of human patients treated with PIs disclosed that their serum PI concentrations reached C_max_ > 1 µM immediately after administration, rapidly declined to nanomolar levels, and remained there for 1 week [18–20]. Hence, we applied 200 nM BTZ and 500 nM CFZ for 1 h and 200 nM MLN2238 for 4 h to simulate the pharmacodynamics and pharmacokinetics of these drugs in human patients.

**Fig. 1:**
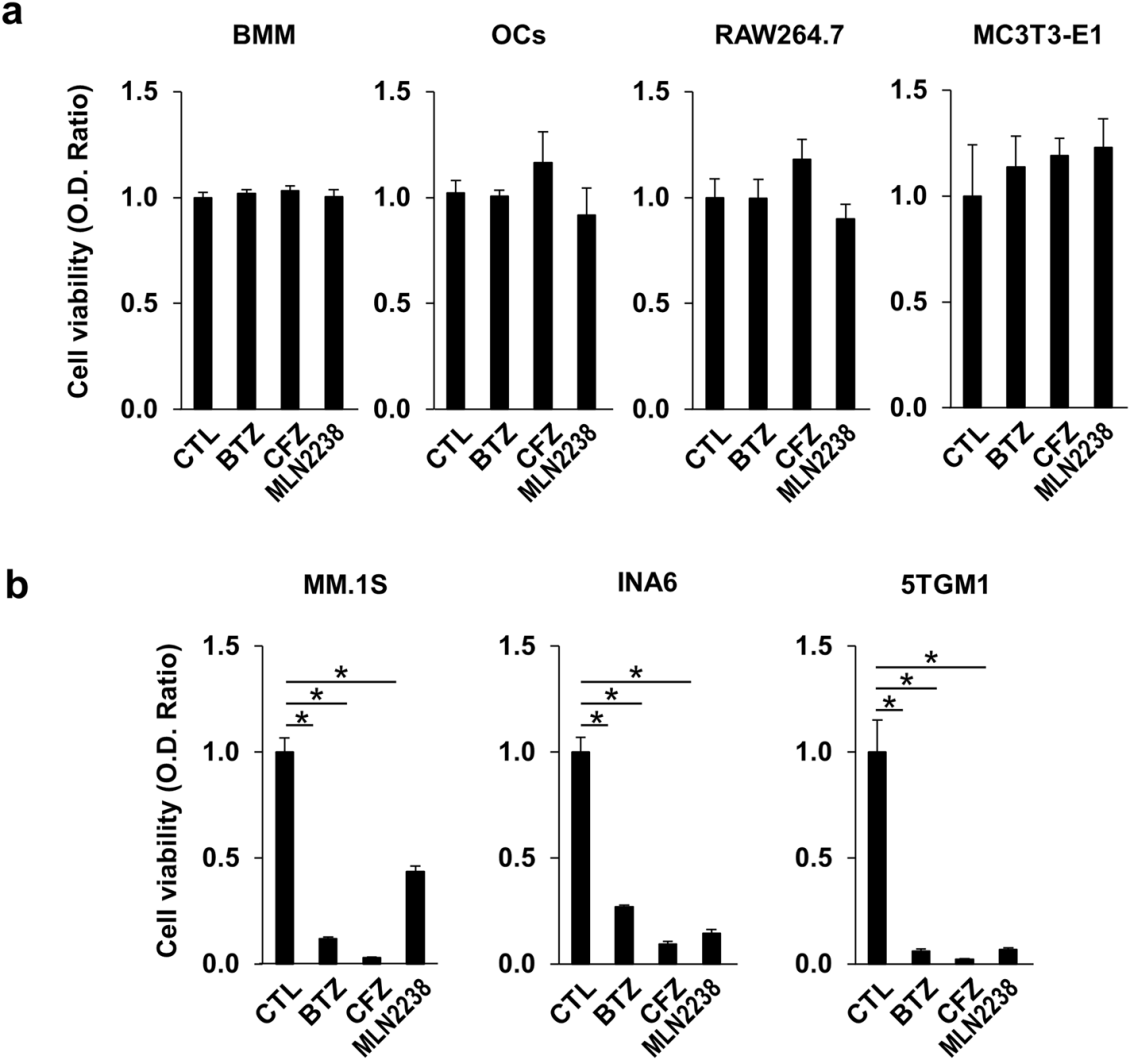
Pulsatile PI treatment induced cell death in MM but not in pre-osteoblasts, bone marrow macrophages, or mature OCs. **a** Bone marrow macrophages (BMMs) and mature osteoclasts (OCs) were generated as described in the Methods section. BMMs, mature OCs, pre-osteoclastic cell line RAW264.7 and pre-osteoblastic cell line MC3T3-E1 were subjected to pulsatile PI treatment as described in the Methods section. The treated cells were washed and then cultured for 24 h. Cell viability was determined by WST-8 assay. Data are means ± SD of six biological replicates. **b** The human MM cell lines MM.1S and INA-6 and the murine 5TGM1 MM cell line were subjected to pulsatile PI treatment as described in the Methods section. The treated cells were washed and then cultured for 24 h. Cell viability was determined by WST-8 assay. Data are means ± SD of six biological replicates. *p < 0.05 by ANOVA followed by Tukey’s test

**Fig. 2:**
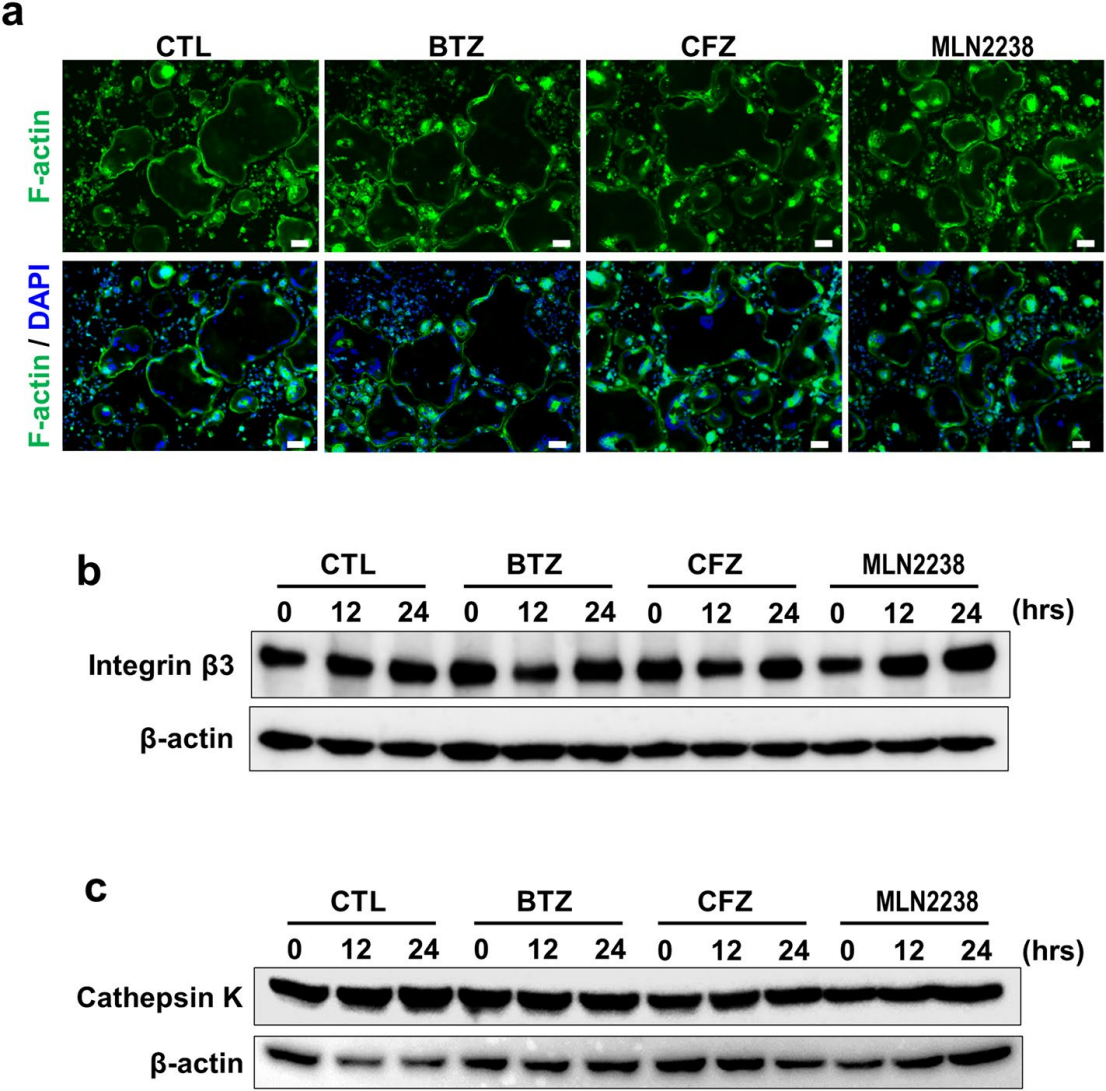
F-actin ring formation was maintained in the large cells subjected to PI pulse treatment. **a** Mature OCs were plated on glass bottom dishes and subjected to PIs as described in the Methods section. The cells were washed and cultured with 50 ng/mL RANKL for 24 h. Then, the cells were fixed and stained with Phalloidin. Scale bar = 100 μm. **b** Mature OCs were subjected to pulsatile PI treatment as described in the Methods section. The cells were washed and then cultured for the indicated time periods. Cell lysates were collected and their integrin β3 protein levels were determined by western blotting. β-actin was a loading control. **c** Mature OCs were subjected to pulsatile PI treatment as described in the Methods section. The cells were washed and then cultured for the indicated time periods. Cell lysates were collected, and their cathepsin K protein levels were determined by western blotting. β-actin was a loading control

We assessed the effects of pulsatile PI treatment on cell viability. Pulsatile treatment with the PIs BTZ, CFZ, and MLN2238 did not reduce the viability of bone marrow macrophages (BMMs), mature OCs, RAW264.7 preosteoclastic cell line or MC3T3-E1 pre-osteoblastic cell line (Fig. 1a). By contrast, pulsatile treatment with these PIs at the same concentrations induced cell death in the MM cell lines MM.1S, INA-6 and 5TGM1 (Fig. 1b). We further evaluated the direct effects of pulsatile PI treatment on mature OCs. F-actin ring formation and integrin β3 expression are vital to mature OC viability and activity. Large F-actin rings are characteristic of mature OCs and they appeared in the control cultures (Fig. 2a). After the pulsatile PI treatment, F-actin ring formation persisted in the large OCs. Integrin β3 was expressed in the mature OCs but did not decrease after the pulsatile PI treatment (Fig. 2b). Cathepsin K is a specific marker of mature OCs. PI pulse treatment did not affect cathepsin K expression in mature OCs (Fig. 2c). The preceding results suggest that OCs but not MM cells resist pulsatile PI treatment.

### Pulsatile PI treatment suppressed RANKL-mediated osteoclastogenesis

**Fig. 3:**
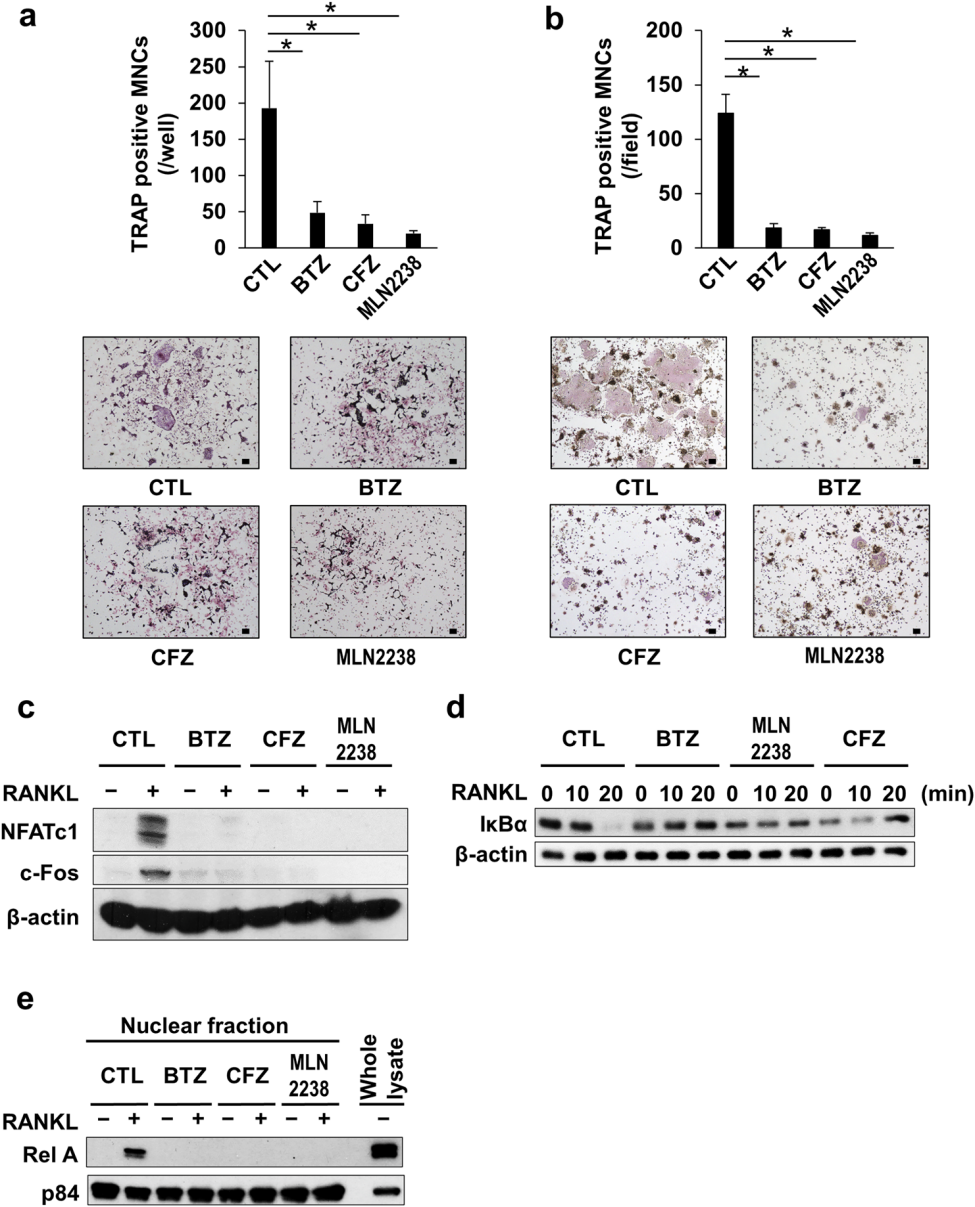
Pulsatile PI treatment suppressed RANKL-induced osteoclastogenesis. BMMs (**a**) or RAW264.7 cells (**b**) were pre-treated with or without BTZ (200 nM) or CFZ (500 nM) for 1 h, or with MLN2238 (200 nM) for 4 h. The BMMs and RAW264.7 cells were washed and cultured with M-CSF (10 ng/mL) and RANKL (50 ng/mL) for 7 and 4 days, respectively. The cells were then fixed and stained with TRAP. TRAP-positive cells containing ≥ 3 nuclei were counted. Data are means ± SD of four biological replicates. *p < 0.05 by ANOVA followed by Tukey’s test. Representative photographs are shown. Original magnification =  × 100. Bar = 100 µm. **c** RAW264.7 cells with or without pulsatile PI pretreatment were cultured with or without RANKL (50 ng/mL) for 24 h. NFATc1 and c-Fos protein levels were determined by western blotting. β-actin was a loading control. **d** RAW264.7 cells with or without pulsatile PI pretreatment were cultured with RANKL (50 ng/mL) for 10 and 20 min. IκBα protein levels were determined by western blotting. β-actin was the loading control. **e** RAW264.7 cells with or without pulsatile PI pretreatment were cultured with RANKL (50 ng/mL) for 24 h. Nuclear extracts were prepared and subjected to western blot analysis with anti-Rel A antibody. p84 was a loading control

RANKL is a critical osteoclastogenesis mediator and is aberrantly upregulated to enhance osteoclastogenesis and bone resorption in MM bone lesions. We explored the effect of the pulsatile PI treatment on OC differentiation. M-CSF and soluble RANKL addition induced TRAP-positive multinucleated cell formation. That is, BMMs (Fig. 3a) and RAW264.7 pre-osteoclastic cells (Fig. 3b) differentiated into OCs. However, the pulsatile PI treatment suppressed TRAP-positive multinucleated cell formation from BMMs and RAW264.7 cells. NFATc1 and c-Fos are critical transcription factors (TFs) in osteoclastogenesis and were upregulated in the RAW264.7 cells subjected to RANKL (Fig. 3c). The pulsatile PI treatment abolished NFATc1 and c-Fos upregulation. RANKL-induced activation of the NF-κB signaling pathway is vital for osteoclastogenesis. RANKL treatment promoted IκBα degradation (Fig. 3d), and nuclear localization of RelA, the NF-κB subunit p65, (Fig. 3e) in RAW264.7 cells. The pulsatile treatment with PIs abrogated these RANKL-induced changes. Therefore, the pulsatile PI treatment might suppress RANKL-induced osteoclastogenesis in part by inhibiting the NF-κB signaling pathway.

### Pulsatile PI treatment suppressed OC bone resorption capacity

**Fig. 4:**
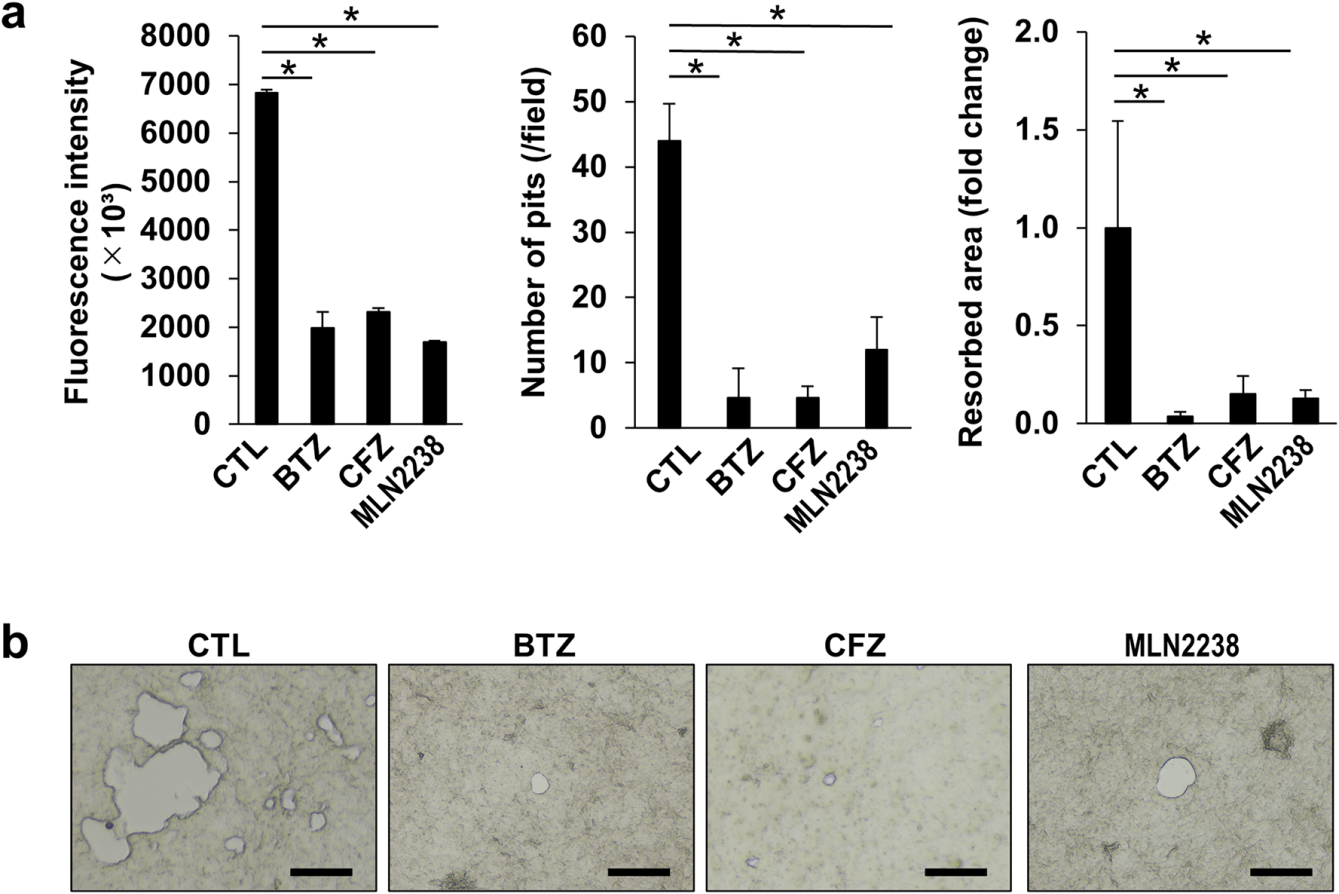
Pulsatile PI treatment suppressed bone resorption by mature OCs. **a** Mature OCs were applied to osteo-assay plates and subjected to PI treatment as described in the Methods section. The cells were washed and cultured in the presence of RANKL (50 ng/mL) for 2 days. The culture media were collected, and the calcium phosphate fluorescence intensity was measured (left). The total number of resorption pits (middle) were counted, and the total areas of resorption pits (right) were analyzed as described in the Methods section. Data are means ± SD of four biological replicate. *p < 0.05 by ANOVA followed by Tukey’s test. **b** Representative photomicrographs of the bone resorption area were shown. Scale bar = 100 μm

Mature OCs were spared from cell death by the pulsatile PI treatment (Fig. 1). Nevertheless, we examined OC function after this treatment. OCs were prepared from BMMs, harvested, and plated on fluoresceinated calcium phosphate-coated dishes. Bone resorption activity was estimated from the pits formed in the fluoresceinated calcium phosphate-coated plates. Supernatant fluorescence intensity in the presence or absence of pulsatile PI treatment was evaluated. The pulsatile PI treatment reduced the relative supernatant fluorescence intensity, the relative number of pits and pit areas formed by the OCs (Fig. 4a). The representative images of the pit formation were shown in Fig. 4b. Thus, the pulsatile PI treatment suppressed bone resorptive activity without impairing OC’s viability.

### Pulsatile PI treatment preserved robust OC-mediated osteoblastogenesis

**Fig. 5:**
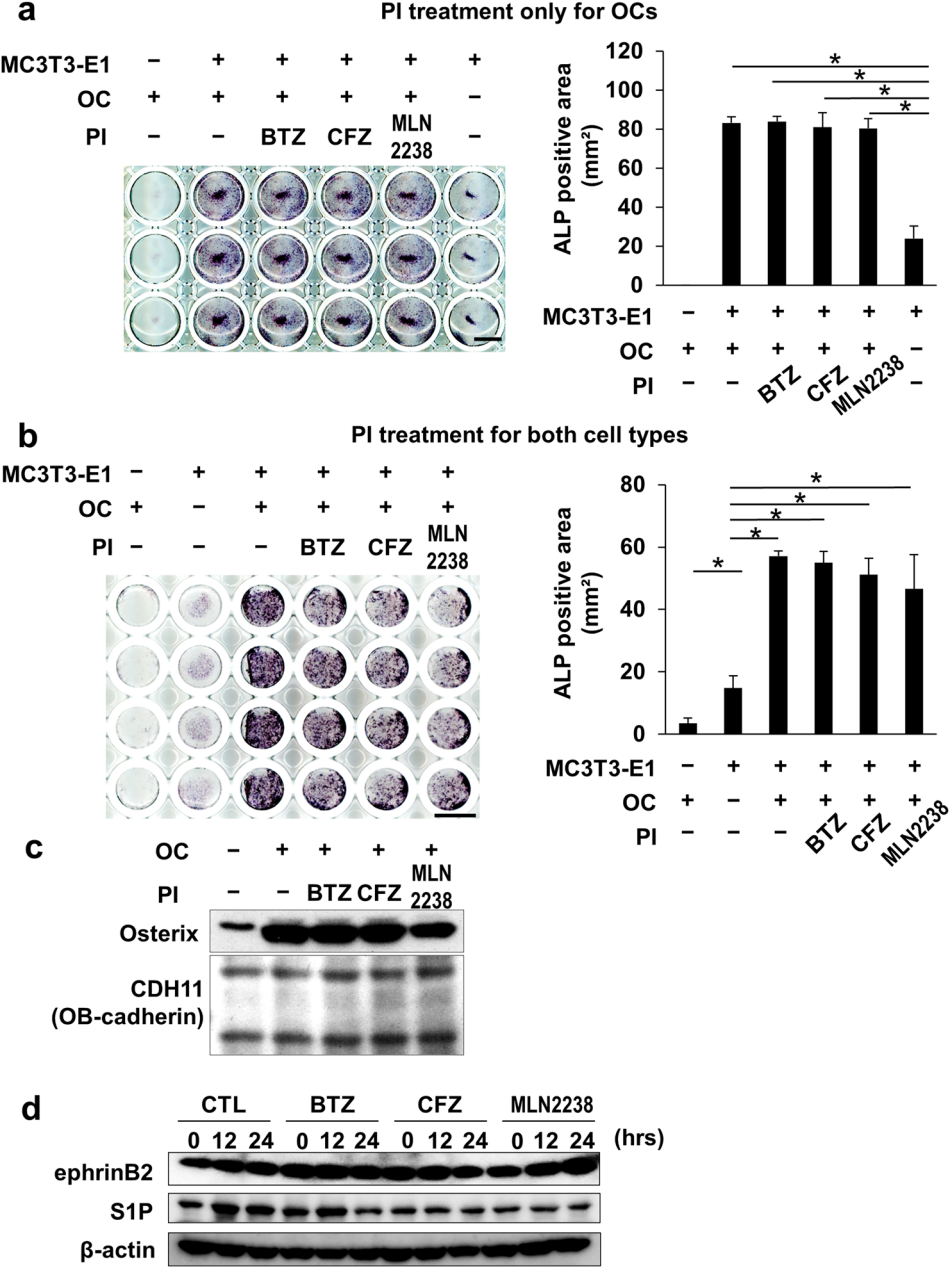
Pulsatile PI treatment retained and exerted OC-mediated bone mineralization in vitro. **a** Mature OCs in the indicated wells were subjected to pulsatile PI treatment as described in the Methods section. The cells were washed and MC3T3-E1 cells were superimposed on them. The two cell types were co-cultured in the osteogenic media for 2 days. OB differentiation was analyzed by ALP staining and the ALP-positive areas (mm^2^) were measured. Data are means ± SD of four biological replicates. *p < 0.05 by ANOVA followed by Tukey’s test. Photomicrographs of the ALP-positive area were shown. Scale bar = 10 mm. **b** MC3T3-E1 cells were superimposed on mature OCs and both types of cells in the indicated wells were subjected to pulsatile PI treatment as described in the Methods section. The cells were then washed and co-cultured in the osteogenic media for 2 days. OB differentiation was analyzed by ALP staining and the ALP-positive areas (mm^2^) were measured. **c** Mature OCs were subjected to pulsatile PI treatment as described in the Methods section. The cells were washed and then MC3T3-E1 cells were superimposed on them and co-cultured in the osteogenic media for 2 days. Cell lysates were collected, and osterix protein levels were determined by western blotting. The osteoblast-specific protein CDH11 (OB-cadherin) was used to estimate the loaded amounts of osteoblast-derived lysates. **d** Mature OCs generated from RAW264.7 pre-osteoclastic cells were subjected to pulsatile PI treatment as described in the Methods section, and then washed and cultured for the indicated periods. Cell lysates were collected and their ephrinB2 and S1P protein levels were determined by western blotting. β-actin was a loading control

OCs induce bone formation at bone resorption sites through OC-derived coupling to osteoblastogenesis. This mechanism depends on the communication between OCs and osteoblasts. We examined OC-derived osteoblastogenesis coupling in response to the pulsatile PI treatment in osteogenic MC3T3-E1 pre-osteoblastic cell cultures. When the MC3T3-E1 pre-osteoblastic cells were cultured alone or plated and co-cultured with the OCs prepared in the culture wells, the OC co-cultured with MC3T3-E1 cells presented substantially enhanced alkaline phosphatase (ALP) activity, an indicator of early osteoblastogenesis. The enhancement of osteoblastogenesis by OCs was maintained either in MC3T3-E1 cells co-cultured with OCs pretreated with the pulsatile treatment with PIs (Fig. 5a) or in both MC3T3-E1 cells and OCs exposed to pulsatile PI treatment after co-culture (Fig. 5b). Osterix is an essential transcription factor for osteoblastogenesis and regarded as a good marker for osteoblastic differentiation. Osterix was upregulated in MC3T3-E1 cells in co-cultured with OCs; however, the pulsatile PIs did not affect the OC-mediated induction of osterix in MC3T3-E1 cells (Fig. 5c). OCs may produce several coupling factors including ephrinB2 and sphingosine-1-phosphate (S1P) which can trigger osteoblastogenesis [25, 26]. Both ephrin B2 and S1P remained upregulated in the OCs following the pulsatile PI treatment (Fig. 5d). These results demonstrate that OCs can potently induce osteoblastogenesis and suggest that pulsatile PI treatment maintains OC-derived coupling to osteoblastogenesis while suppressing OC’s bone resorption activity and inducing MM cell death.

## Discussion

In clinical practice, patients treated with PIs achieve a good response with very good partial response or more have been demonstrated to resume significant bone formation preferentially in osteoclastic bone destructive MM lesions without hyperostosis in normal bones, which is a therapeutic merit unique to PIs [14–17]. Patients with MM exhibiting bone formation tend to show a better and prolonged reduction of MM tumor. PIs have been demonstrated to transcriptionally upregulate Runx2 [27, 28] which is a critical TF in early osteoblastogenesis and cause the accumulation of various mediators of the β-catinine, Osterix/Sp7, and ATF4 signaling pathways responsible for osteoblastogenesis by blockading their proteasomal degradation [17, 29, 30]. PIs also suppressed DKK1 production in the bone marrow microenvironment and sclerostin production in osteocytes [17, 31]. Bortezomib can protect bone loss in non-tumorous animal osteoporotic models [28, 32]. With enough reduction of MM tumor cells, PIs are thought to enhance the levels of critical osteoblastogenesis-related TFs such as Runx2 and ATF4 [27, 30], and thereby rebuild bone in MM. The direct effects of PIs on osteoblastogenesis have been demonstrated mainly by in vitro cultures of osteoblastic lineage cells with long-term exposure to relatively low concentrations of PIs. Continuous treatment with PIs at higher concentrations (ex. Bortezomib over 10 nM) rather hampered in vitro osteoblastogenesis [30]. However, MM patients treated with PIs exhibit pulsatile high PI concentrations in their sera. Only with the direct anabolic actions of PIs, it is hard to explain rapid and selective bone recovery in bone-defective lesions without hyperostosis in normal bones in good responders to PIs.

In terms of the experimental conditions of bortezomib or carfilzomib exposure, we basically followed the previous paper with experiments modeling the anticipated in vivo pharmacokinetics of drug exposure in which MM cells were treated with pulsatile treatment for one hour of bortezomib or carfilzomib at concentrations mainly between 100 and 300 and between 100 and 500 nM, respectively [33]. However, carfilzomib can be currently used with 30-min iv infusion at 70 mg/m^2^ which makes much higher maximum observed plasma concentrations (Cmax) compared with those with the administration at 27 and 56 mg/m^2^. Therefore, we set experimental conditions with one-hour treatment of carfilzomib at 500 nM, the highest concentration in the previous paper [33]. Ixazomib is an oral agent and thus its inter-patient variability in Cmax is wide. Maximum drug concentration time from oral intake (Tmax) was also widely distributed over 3 h. Therefore, blood concentrations of ixazomib can reach 200 nM for 4 h in a certain portion of patients after taking ixazomib orally. By taking into consideration the wide distribution of Cmax and Tmax with long T1/2, we set 200 nM for 4 h as an experimental condition for MLN2238 exposure to mimic a PK profile in patients with good bioavailability of this drug. Here, we examined the effects of short-term pulsatile treatment with BTZ (200 nM) and CFZ (500 nM) for 1 h and MLN2238 (200 nM) for 4 h to simulate the pharmacokinetic profile of PIs in MM patients treated with them. The pulsatile PI treatment suppressed the osteoclastogenesis of OC precursors and bone resorption by mature OCs without impairing OC viability. We previously reported that OCs and their precursors resisted the ROS-inducible anti-cancer agent doxorubicin and utilized ROS in their own differentiation and activation [34]. Doxorubicin enhanced RANKL-induced osteoclastogenesis by inducing NFATc1 which is a critical TF in early osteoclastogenesis. The results of the present study suggested that OCs and their precursors resist pulsatile PI treatment at high concentrations as the cells maintained their viability and F-actin ring formation. By contrast, MM cells underwent cell death under the same treatments. Unlike doxorubicin, however, the pulsatile PI treatment suppressed RANKL-induced NFATc1 expression and thereby osteoclastic differentiation, and repressed bone resorption by mature OCs.

After OCs resorb damaged or old bone during normal bone remodeling, they locally produce multiple coupling factors, enhance osteoblastogenesis, and replace the resorbed bone with new bone matrix [5]. Thus, OCs are vital for potent osteogenic activity and effective bone formation. Here, osteoblastogenesis was markedly induced in the presence of OCs (Fig. 5). Hence, there was a coupling between OCs and osteoblasts resulting in OC-induced osteoblastogenesis or bone formation. Robust OC-induced osteoblastogenesis persisted in response to the pulsatile PI treatment. MM cells overproduce inhibitory factors for osteoblastogenesis including the soluble Wnt antagonists DKK1 and sFRP family members, and we previously reported that MM cell-derived conditioned media suppress osteoblastogenesis [7, 35]. In active MM bone lesions where MM cells and OCs accumulate, osteoblastogenesis is markedly suppressed. However, substantial MM cell reduction with PIs allows OC-mediated osteoblastogenesis through elimination of the MM cell-derived inhibitors. Therefore, OC-driven osteoblastogenesis might be the predominant mechanism by which bone is restored in bone-defective lesions where OCs reside under PI-based treatment. In patients who respond favorably to PIs, PIs may sufficiently reduce MM cell-derived inhibitors for osteoblastogenesis to facilitate OC-driven bone formation coupling and the suppression of OC differentiation and activity.

Suppression of OC’s bone resorptive activity with retaining OC viability has been demonstrated to efficiently increase bone formation in patients with osteoporosis under treatment with cathepsin K inhibitors [36]. Cathepsin K inhibitors inhibit the enzymatic degradation of bone matrix by cathepsin K secreted from mature active OCs without affecting OC viability. Although cathepsin K inhibitors do not directly affect osteoblastogenesis, treatment with cathepsin K inhibitors has been demonstrated to robustly enhance bone formation [37, 38]. Cathepsin K inhibition might permit OCs to enhance osteoblastogenesis by inducing coupling factor production by OCs while suppressing OC bone resorption [39]. As observed in a randomized, double-blind, multicenter phase 3 study of denosumab compared with zoledronic acid in the treatment of bone disease in subjects with newly diagnosed MM with at least one image-documented bone lesion, 60% of new skeletal related events on study occurred within the first 3 months [40], suggesting the presence of active OCs after administration of these potent anti-bone resorptive agents. Thus, abundant active OCs persist in osteoclastic bone lesions in the early course of anti-MM treatment under repeated denosumab or zoledronic acid administration. By analogy with the anabolic effects of cathepsin K inhibitors, the pulsatile PI treatment suppressed bone resorption by mature OCs in MM bone lesions while permitting viable OCs to couple to osteoblastogenesis in early responders with substantially reducing MM cell-derived inhibitors for osteoblastogenesis. Taken together, the mechanistic view of OC-driven coupling to osteoblastogenesis explains why bone regeneration preferentially occurs in bone-destructive lesions in good responders during the early course of PI-based treatment.

## Data Availability

All data in this article is available through personal communication with the the corresponding author.
